# Crystal structure of di­ammonium bis­[tris­(oxamide dioxime-κ^2^ *N*,*N*′)nickel(II)] bis­[tris­(oxalato-κ^2^ *O*,*O*′)chromate(III)] 6.76-hydrate

**DOI:** 10.1107/S2056989020013390

**Published:** 2020-10-09

**Authors:** Yves Alain Mbiangué, Alfred Bijvédé, Patrice Kenfack Tsobnang, Emmanuel Wenger, Claude Lecomte

**Affiliations:** a Chemistry Department, Higher Teachers’ Training College, University of Maroua, PO Box 55, Maroua, Cameroon; b Chemistry Department, Faculty of Science, University of Maroua, PO Box 814, Maroua, Cameroon; c Chemistry Department, Faculty of Science, University of Dschang, PO Box 67, Dschang, Cameroon; d Université de Lorraine, CNRS, CRM^2^, F54000, Nancy, France

**Keywords:** crystal structure, tris­(oxamide dioxime)nickel(II), tris­(oxalato)chromate(III), hydrogen-bonded network, supra­molecular assembly

## Abstract

The asymmetric unit of the title compound, (NH_4_)_2_[Ni(C_2_H_6_N_4_O_2_)_3_]_2_[Cr(C_2_O_4_)_3_]_2_·6.76H_2_O, comprises two NH_4_^+^ cations, two [Ni(C_2_H_6_N_4_O_2_)_3_]^2+^ cations and two [Cr(C_2_O_4_)_3_]^3–^ anions, as well as eight water mol­ecules of crystallization of which only one is fully occupied. In the cationic and anionic complexes, the central atoms (Ni^II^ and Cr^III^) are each surrounded by three bidentate ligands (N-chelating oxamide dioxime and O-chelating oxalate, respectively), resulting in distorted octa­hedral coordination spheres. In the crystal, O—H⋯O hydrogen bonds between the oxamide dioxime ligands as donor groups and the oxalate ligands as acceptor groups alternately connect the cationic and anionic complexes into infinite pillars extending parallel to [100]. Moreover, N—H⋯O hydrogen bonds between the same ligands connect neighboring pillars, thus delineating channels that accommodate the charge-balancing NH_4_^+^ cations as well as the water mol­ecules of crystallization. Although the H atoms could not be localized for these two species, the corresponding N⋯O and O⋯O distances indicate hydrogen bonds of medium strength.

## Abstract 2

In the structure of the title compound, [Ni(C_2_H_6_N_4_O_2_)_3_]^2+^ cations and [Cr(C_2_O_4_)_3_]^3–^ anions are ordered alternately into negatively charged hydrogen-bonded pillars running parallel to the *a* axis. These pillars delimit channels accommodating the charge-balancing NH_4_^+^ cations as well as the water mol­ecules of crystallization.

## Chemical context   

Tris(oxalato)metallate(III) complex anions, [*M* ^III^(C_2_O_4_)_3_]^3–^, are versatile building blocks for the design of new mol­ecule-based materials with inter­esting magnetic, electrical and optical properties (Coronado *et al.*, 2000 ▸). Through coordin­ation bonds with a variety of metallic ions, these anions can act as ligands, forming various one-, two- and three-dimensional polymeric networks (Pardo *et al.*, 2012 ▸; Decurtins *et al.*, 1998 ▸). Moreover, in the presence of appropriate hydrogen-donor groups, they can act as hydrogen-bond acceptors resulting in a multitude of hydrogen-bonded networks (Kenfack Tsobnang *et al.*, 2014 ▸; Muzioł *et al.*, 2011 ▸; Zhuge *et al.*, 2010 ▸; Borel *et al.*, 2009 ▸). When [*M* ^III^(C_2_O_4_)_3_]^3–^ anions are combined with triply charged tris-bidentate complex cations of *D*_3_ symmetry in which the ligating atoms are all bonded to H atoms or OH groups, they build infinite neutral pillars of alternating complex cations and anions that leave channels in the structure (Bélombé *et al.*, 2009 ▸; Hua *et al.*, 2001 ▸; Kuroda, 1991 ▸). If functional species (such as spin-crossover or photochromic complexes) are inserted into such voids, inter­esting properties of the resulting material can be expected, similar to what has been achieved with oxalate-based two-dimensional polymeric networks (Clemente-León *et al.*, 2011 ▸). A convenient way of forcing additional species into the channels would be by designing compounds with charged, instead of neutral, pillars. In this way, the charge-balancing species could only reside in the channels. This strategy proved successful by combining tris­(oxalato)chromate(III) anions, [Cr(C_2_O_4_)_3_]^3–^, with tris(oxamide dioxime)nickel(II) cations, [Ni(C_2_H_6_N_4_O_2_)_3_]^2+^, the charge-balancing species being K^+^ and H_3_O^+^ (Mbiangué *et al.*, 2012 ▸). An attempt to insert NH_4_^+^ (a proton carrier) into such channels led to (NH_4_)_2_[Ni(C_2_H_6_N_4_O_2_)_3_]_2_[Cr(C_2_O_4_)_3_]_2_·6.76H_2_O (**I**). Herein, we report its structure.
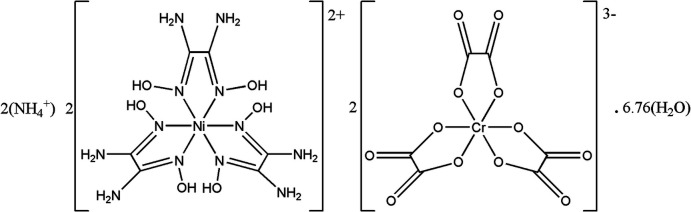


## Structural commentary   

**Figure 1:**
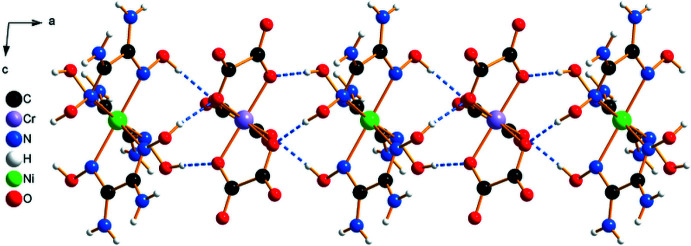
Arrangement of alternating [Ni(C_2_H_6_N_4_O_2_)_3_]^2+^ cations and [Cr(C_2_O_4_)_3_]^3−^ anions into pillars in the structure of (**I**), viewed along [010]. Dashed lines indicate hydrogen bonds.

**Figure 3:**
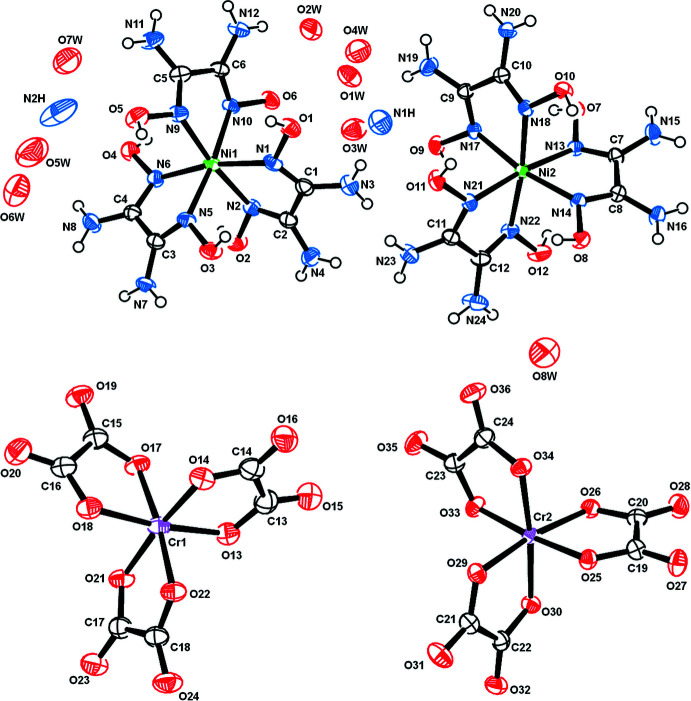
The mol­ecular components of the asymmetric unit of (**I**), showing the atom-numbering scheme and displacement ellipsoids at the 50% probability level.

The structure of (**I**) is made up of infinite negatively charged pillars of alternating [Ni(C_2_H_6_N_4_O_2_)_3_]^2+^ cations and [Cr(C_2_O_4_)_3_]^3–^ anions. The pillars run parallel to [100] and delimit channels containing the charge-compensating cations, NH_4_^+^, as well as the water mol­ecules of crystallization (Figs. 1 ▸, 2 ▸). The mol­ecular components of the asymmetric unit are depicted in Fig. 3 ▸. For each metal, two crystallographically independent sites (Ni1 and Ni2 and Cr1 and Cr2, respectively) are present. All of these sites are coordinated in the form of distorted octa­hedra by six imino N atoms from three bidentate oxamide dioxime ligands (for the nickel sites) and six O atoms from three bidentate oxalate ligands (for the chromium sites). The resulting complexes are chiral. Within a pillar, all the metallic sites have the same chirality, either Δ or Λ. Thus, each pillar is chiral but related to another pillar in the crystal through an inversion center. The Ni—N bond lengths range from 2.051 (3) to 2.097 (3) Å and the Cr—O bond lengths, from 1.947 (3) to 1.983 (3) Å (Table 1 ▸). Within a pillar, the Ni1⋯Cr1 distances alternate between 4.8897 (8) and 4.9170 (8) Å, and the Ni2⋯Cr2 distances between 4.8743 (7) and 4.9323 (7) Å.

## Supra­molecular features   

**Figure 4:**
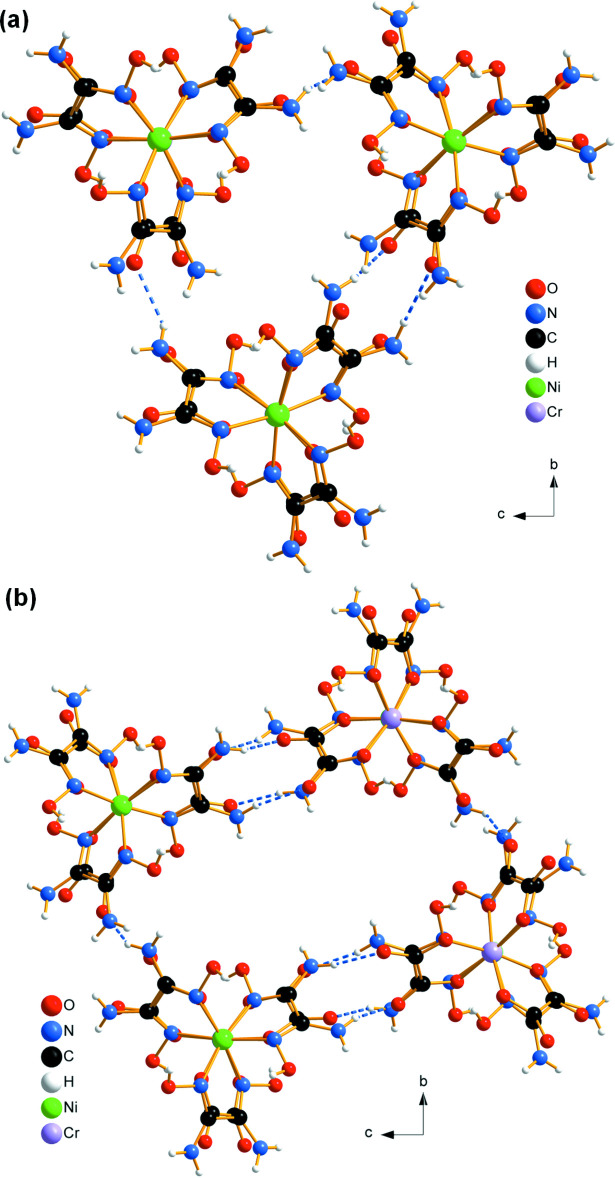
The connection of three (*a*) and four (*b*) neighboring pillars through hydrogen bonds (dashed lines) in the structure of (**I**).

In the crystal, extensive N—H⋯O and O—H⋯O hydrogen-bonding inter­actions are observed (Table 2 ▸). Neighboring metal complexes are linked by inter­molecular O—H⋯O hydrogen bonds between three hydroxyl groups from the oxamide dioxime ligands as donor groups and three ligating O atoms from the oxalate ligands as acceptors. These inter­actions connect the metal complexes into pillars running parallel to the [100] direction (Fig. 1 ▸). Adjacent pillars are further linked to each other through inter­molecular N—H⋯O hydrogen bonds involving the amino groups from the oxamide dioxime ligands as donor groups and the non-coordinating O atoms from the oxalate ligands as acceptor groups. The formed pillars delimit two types of channel propagating parallel to [100] (Fig. 4 ▸). Five of these N—H⋯O hydrogen bonds are bifurcated: N4—H4*B*⋯(O19^iii^,O35^i^), N7—H7*B*⋯(O35^iii^,O36^iii^), N15—H15*B*⋯(O31^viii^,O32^viii^), N19—H19*B*⋯(O27^vi^,O28^vi^) and N20—H20*B*⋯(O27^vi^,O28^vi^) (symmetry codes refer to Table 2 ▸). There are two N—H⋯O hydrogen bonds between two amino groups from the oxamide dioxime ligands as donor groups and two water mol­ecules (O1*W* and O6*W*) as acceptor groups. There are also numerous hydrogen-bonding inter­actions involving the water mol­ecules and the ammonium cations (Table 2 ▸). Together, these hydrogen-bonding inter­actions lead to a three-dimensional hydrogen-bonded network. Although the H atoms could not be localized for the ammonium cations and water mol­ecules of crystallization, the corresponding N⋯O and O⋯O distances (Table 2 ▸) indicate hydrogen bonds of medium strength.

## Database survey   

A search of the Cambridge Structural Database (CSD version 5.41, August 2020 update; Groom *et al.*, 2016 ▸) for tris-bidentate transition metal complexes with five membered chelate rings and only N-donor atoms gave 5914 hits. A search for similar complexes but with O-donor atoms gave 1009 hits. A combined search with the two previous queries gave 77 hits. A close examination of the latter structures revealed that only four of them contain hydrogen-bonded pillars of alternating cations and anions with *D*_3_ symmetry. Their CSD refcodes are RUPGEP (Bélombé *et al.*, 2009 ▸), IFOCEL and IFOCIP (Hua *et al.*, 2001 ▸), and SOZFIW (Kuroda, 1991 ▸). A related compound of formula (H_3_O)[K(H_2_O)_3_][Ni(C_2_H_6_N_4_O_2_)_3_]_2_[Cr(C_2_O_4_)_3_]_2_·3H_2_O, absent from the CSD, was reported a few years ago (Mbiangué *et al.*, 2012 ▸).

## Synthesis and crystallization   

The two precursor salts, (NH_4_)_3_[Cr(C_2_O_4_)_3_]·3H_2_O (Bailar & Jones, 1939 ▸) and [Ni(C_2_H_6_N_4_O_2_)_3_]SO_4_·5H_2_O (Bélombé *et al.*, 2008 ▸), were synthesized as described in the literature. The title compound was prepared as follows: finely powdered [Ni(C_2_H_6_N_4_O_2_)_3_]SO_4_·5H_2_O (0.18 g, 0.30 mmol) was added in successive small portions to an aqueous solution (20 ml) of (NH_4_)_3_[Cr(C_2_O_4_)_3_]·3H_2_O (0.13 g, 0.31 mmol) acidified with two drops of sulfuric acid. The resulting violet mixture was stirred at room temperature (303 K) for 45 min and then filtered. The filtrate was left for evaporation. After one day, violet single crystals were harvested. Upon drying, these crystals lost their brightness, suggesting a possible dehydration.

## Refinement   

Crystal data, data collection and structure refinement details are summarized in Table 3 ▸. All hydrogen atoms of the cationic complex were located in difference-Fourier maps but were finally placed in geometrically idealized positions with *U*_iso_(H) = 1.2*U*_eq_(N) and *U*_iso_(H) = 1.5*U*_eq_(O). The assignment of water O atoms and ammonium N atoms was not straightforward. Hence, ten isolated peaks with significant electron densities (between 3.46 and 9.45 e^−^Å^−3^) were first modeled as N atoms. Their site occupancies were subsequently refined freely. Two of these ten N atoms then had site occupancies inferior but close to unity (0.98 and 0.99). Finally, taking into consideration the electroneutrality of the crystal, the assignment of the aforementioned two N atoms (labeled as N1*H* and N2*H*) was assumed to be correct and their site occupancies were fixed to 1. The remainder of the alleged N atoms were finally treated as water O atoms. The site occupancies of these O atoms were fixed to 1 for one of them (O1*W*) and refined to 0.797 (12), 0.840 (11), 0.835 (11), 0.692 (14), 0.878 (14), 0.909 (13) and 0.812 (14), for the seven others (O2*W*–O8*W*). The hydrogen atoms of the ammonium ions and water mol­ecules could not be found in difference-Fourier maps, but they were included in the final formula.

## Supplementary Material

Crystal structure: contains datablock(s) I. DOI: 10.1107/S2056989020013390/wm5586sup1.cif

Structure factors: contains datablock(s) I. DOI: 10.1107/S2056989020013390/wm5586Isup2.hkl

CCDC reference: 2032214

Additional supporting information:  crystallographic information; 3D view; checkCIF report

## Figures and Tables

**Figure 2:**
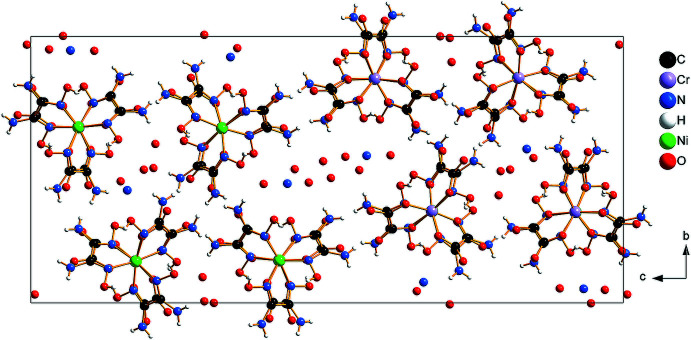
A view along [100] of the crystal packing of (**I**), illustrating the orientation of the complex ions in an eclipsed configuration within each pillar as well as the channels between the pillars.

**Table 1:** Selected bond lengths (Å)

Cr1—O14	1.947 (3)	Ni1—N2	2.055 (3)
Cr1—O13	1.962 (3)	Ni1—N6	2.059 (3)
Cr1—O17	1.969 (3)	Ni1—N5	2.076 (3)
Cr1—O22	1.975 (2)	Ni1—N9	2.083 (3)
Cr1—O21	1.977 (3)	Ni1—N1	2.083 (3)
Cr1—O18	1.983 (3)	Ni1—N10	2.097 (3)
Cr2—O25	1.954 (2)	Ni2—N13	2.051 (3)
Cr2—O33	1.959 (2)	Ni2—N17	2.067 (3)
Cr2—O29	1.963 (2)	Ni2—N14	2.071 (3)
Cr2—O34	1.968 (2)	Ni2—N21	2.083 (3)
Cr2—O30	1.973 (2)	Ni2—N18	2.086 (3)
Cr2—O26	1.979 (2)	Ni2—N22	2.086 (3)

**Table 2:** Hydrogen-bond geometry (Å, °)

*D*—H⋯*A*	*D*—H	H⋯*A*	*D*⋯*A*	*D*—H⋯*A*
N3—H3*A*⋯O9	0.88	2.42	2.863 (4)	112
N3—H3*B*⋯O35^i^	0.88	2.52	3.387 (4)	168
N4—H4*A*⋯O2^ii^	0.88	2.13	2.811 (4)	134
N4—H4*B*⋯O19^iii^	0.88	2.21	2.954 (4)	142
N4—H4*B*⋯O35^i^	0.88	2.65	3.190 (4)	121
N7—H7*A*⋯O16^iii^	0.88	2.32	3.183 (4)	167
N7—H7*B*⋯O35^iii^	0.88	2.36	3.198 (4)	159
N7—H7*B*⋯O36^iii^	0.88	2.58	3.228 (4)	131
N8—H8*A*⋯O6*W*	0.88	2.34	3.135 (6)	150
N8—H8*B*⋯O36^iii^	0.88	2.06	2.932 (4)	169
N11—H11*B*⋯O28^iv^	0.88	2.14	2.913 (4)	146
N12—H12*A*⋯O7^v^	0.88	2.56	3.170 (4)	128
N12—H12*B*⋯O32^vi^	0.88	2.18	3.007 (4)	155
N15—H15*A*⋯O1*W* ^vii^	0.88	2.09	2.948 (4)	166
N15—H15*B*⋯O31^viii^	0.88	2.25	3.057 (4)	152
N15—H15*B*⋯O32^viii^	0.88	2.34	3.020 (4)	134
N16—H16*A*⋯O23^ix^	0.88	2.30	3.129 (4)	157
N16—H16*B*⋯O32^viii^	0.88	2.43	3.295 (4)	167
N19—H19*A*⋯O1*W*	0.88	2.07	2.912 (4)	159
N19—H19*B*⋯O27^vi^	0.88	2.08	2.924 (4)	160
N19—H19*B*⋯O28^vi^	0.88	2.59	3.210 (4)	128
N20—H20*A*⋯O24^viii^	0.88	2.38	3.215 (4)	158
N20—H20*B*⋯O27^vi^	0.88	2.52	3.086 (4)	123
N20—H20*B*⋯O28^vi^	0.88	2.52	3.383 (4)	168
N23—H23*A*⋯O16	0.88	2.17	2.858 (4)	135
N23—H23*B*⋯O19^iii^	0.88	2.15	2.983 (4)	159
N24—H24*A*⋯N2*H* ^ix^	0.88	2.57	3.394 (8)	156
N24—H24*B*⋯O20^iii^	0.88	2.25	3.116 (4)	170
O1—H1⋯O22	0.84	1.91	2.750 (4)	176
O2—H2⋯O17^i^	0.84	1.81	2.644 (4)	176
O3—H3⋯O14	0.84	1.90	2.730 (4)	171
O4—H4⋯O21^i^	0.84	1.89	2.726 (4)	172
O5—H5⋯O18	0.84	1.81	2.647 (4)	173
O6—H6⋯O13^i^	0.84	1.90	2.727 (4)	171
O7—H7⋯O30^i^	0.84	1.87	2.698 (3)	167
O8—H8⋯O34	0.84	1.97	2.775 (3)	159
O9—H9⋯O33^i^	0.84	1.84	2.679 (3)	177
O10—H10⋯O25	0.84	1.81	2.642 (3)	171
O11—H11⋯O29	0.84	1.85	2.681 (3)	172
O12—H12⋯O26^i^	0.84	1.86	2.702 (3)	177
O15⋯N1*H*			2.737 (5)	
O20⋯O5*W*			2.740 (7)	
O23⋯O7*W*			2.810 (5)	
O24⋯O2*W*			2.840 (5)	
O36⋯O8*W*			2.797 (6)	
O1*W*⋯N1*H* ^i^			2.856 (5)	
O3*W*⋯O4*W*			2.705 (6)	
O5*W*⋯O8*W* ^x^			2.817 (11)	
O6*W*⋯N2*H* ^xi^			2.674 (11)	
O6*W*⋯O6*W* ^xi^			2.723 (11)	
O6*W*⋯O8*W* ^iv^			2.779 (8)	
O7*W*⋯O8*W* ^x^			2.729 (8)	

Symmetry codes: (i) 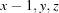; (ii) 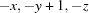; (iii) 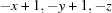; (iv) 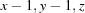; (v) 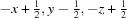; (vi) 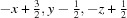; (vii) 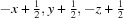; (viii) 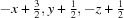; (ix) 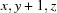; (x) 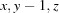; (xi) 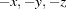.

**Table 3:** Experimental details

Crystal data	
Chemical formula	(NH_4_)_2_[Ni(C_2_H_6_N_4_O_3_)_3_][Cr(C_2_O_4_)_3_]·6.76H_2_O
*M*_r_	1616.12
Crystal system, space group	Monoclinic, *P*2_1_/*n*
Temperature (K)	110
*a*, *b*, *c* (Å)	9.8065 (3), 16.6719 (4), 37.2296 (9)
β (°)	95.562 (3)
*V* (Å^3^)	6058.1 (3)
*Z*	4
Radiation type	Cu *K*α
μ (mm^−1^)	4.74
Crystal size (mm)	0.22 × 0.10 × 0.05

Data collection	
Diffractometer	Rigaku Supernova, Dual, Cu at zero, Atlas
Absorption correction	Gaussian (*CrysAlis PRO*; Rigaku OD, 2015 ▸)
*T*_min_, *T*_max_	0.503, 1.000
No. of measured, independent and observed [*I* > 2σ(*I*)] reflections	97200, 12706, 11294
*R*_int_	0.063
(sin θ/λ)_max_ (Å^−1^)	0.632

Refinement	
*R*[*F* ^2^ > 2σ(*F* ^2^)], *wR*(*F* ^2^), *S*	0.060, 0.178, 1.06
No. of reflections	12706
No. of parameters	903
H-atom treatment	H-atom parameters constrained
Δρ_max_, Δρ_min_ (e Å^−3^)	1.42, −1.03

Computer programs: *CrysAlis PRO* (Rigaku OD, 2015 ▸), *SHELXT* (Sheldrick, 2015*a* ▸), *SHELXL2018/3* (Sheldrick, 2015*b* ▸), *ORTEP-3 for Windows* (Farrugia, 2012 ▸) and *DIAMOND* (Brandenburg & Putz, 2018 ▸), *WinGX* (Farrugia, 2012 ▸) and *publCIF* (Westrip, 2010 ▸).

## supplementary crystallographic information

### Crystal data

**Table:**

(NH_4_)_2_[Ni(C_2_H_6_N_4_O_3_)_3_][Cr(C_2_O_4_)_3_]·6.76H_2_O	*F*(000) = 3319
*M**_r_* = 1616.12	*D*_x_ = 1.772 Mg m^−^^3^
Monoclinic, *P*2_1_/*n*	Cu *K*α radiation, λ = 1.54184 Å
*a* = 9.8065 (3) Å	Cell parameters from 25623 reflections
*b* = 16.6719 (4) Å	θ = 3.6–76.1°
*c* = 37.2296 (9) Å	µ = 4.74 mm^−^^1^
β = 95.562 (3)°	*T* = 110 K
*V* = 6058.1 (3) Å^3^	Block, light violet
*Z* = 4	0.22 × 0.10 × 0.05 mm

### Data collection

**Table:**

Rigaku Supernova, Dual, Cu at zero, Atlas diffractometer	12706 independent reflections
Radiation source: micro-focus sealed X-ray tube	11294 reflections with *I* > 2σ(*I*)
Detector resolution: 10.4508 pixels mm^-1^	*R*_int_ = 0.063
ω scans	θ_max_ = 77.1°, θ_min_ = 3.6°
Absorption correction: gaussian (CrysAlisPro; Rigaku OD, 2015)	*h* = −11→12
*T*_min_ = 0.503, *T*_max_ = 1.000	*k* = −20→21
97200 measured reflections	*l* = −46→44

### Refinement

**Table:**

Refinement on *F*^2^	Secondary atom site location: difference Fourier map
Least-squares matrix: full	Hydrogen site location: inferred from neighbouring sites
*R*[*F*^2^ > 2σ(*F*^2^)] = 0.060	H-atom parameters constrained
*wR*(*F*^2^) = 0.178	*w* = 1/[σ^2^(*F*_o_^2^) + (0.087*P*)^2^ + 14.3935*P*] where *P* = (*F*_o_^2^ + 2*F*_c_^2^)/3
*S* = 1.06	(Δ/σ)_max_ = 0.001
12706 reflections	Δρ_max_ = 1.42 e Å^−^^3^
903 parameters	Δρ_min_ = −1.03 e Å^−^^3^
0 restraints	Extinction correction: SHELXL-2018/3 (Sheldrick, 2015b), Fc^*^=kFc[1+0.001xFc^2^λ^3^/sin(2θ)]^-1/4^
Primary atom site location: structure-invariant direct methods	Extinction coefficient: 0.00017 (3)

### Special details

**Table:**

Geometry. All esds (except the esd in the dihedral angle between two l.s. planes) are estimated using the full covariance matrix. The cell esds are taken into account individually in the estimation of esds in distances, angles and torsion angles; correlations between esds in cell parameters are only used when they are defined by crystal symmetry. An approximate (isotropic) treatment of cell esds is used for estimating esds involving l.s. planes.

### Fractional atomic coordinates and isotropic or equivalent isotropic displacement parameters (Å^2^)

**Table:**

	*x*	*y*	*z*	*U*_iso_*/*U*_eq_	Occ. (<1)
Cr1	0.68174 (6)	0.34082 (4)	0.08081 (2)	0.02787 (16)	
Cr2	0.97595 (5)	0.84426 (3)	0.17692 (2)	0.01784 (13)	
Ni1	0.18292 (6)	0.34083 (3)	0.08024 (2)	0.02253 (15)	
Ni2	0.47905 (5)	0.84438 (3)	0.17732 (2)	0.01840 (14)	
C1	0.2393 (4)	0.5086 (2)	0.09426 (9)	0.0286 (7)	
C2	0.1288 (4)	0.5065 (2)	0.06463 (9)	0.0271 (7)	
C3	0.1932 (3)	0.3064 (2)	0.00496 (9)	0.0248 (6)	
C4	0.0974 (3)	0.2453 (2)	0.01735 (9)	0.0256 (7)	
C5	0.2559 (3)	0.2231 (2)	0.13544 (10)	0.0292 (7)	
C6	0.1685 (3)	0.2812 (2)	0.15249 (9)	0.0248 (6)	
C7	0.4432 (3)	0.98703 (19)	0.21879 (9)	0.0222 (6)	
C8	0.5383 (3)	1.01223 (19)	0.19201 (9)	0.0209 (6)	
C9	0.4447 (3)	0.69710 (19)	0.21655 (9)	0.0221 (6)	
C10	0.5546 (3)	0.74110 (19)	0.23883 (8)	0.0209 (6)	
C11	0.4931 (3)	0.7777 (2)	0.10617 (9)	0.0235 (6)	
C12	0.3911 (3)	0.8438 (2)	0.10062 (9)	0.0261 (7)	
C13	0.7688 (4)	0.4982 (2)	0.09099 (11)	0.0367 (8)	
C14	0.6267 (4)	0.5024 (2)	0.06938 (10)	0.0345 (8)	
C15	0.7034 (4)	0.2972 (2)	0.00937 (10)	0.0321 (8)	
C16	0.5863 (4)	0.2458 (2)	0.02192 (10)	0.0333 (8)	
C17	0.7617 (4)	0.2274 (2)	0.13340 (10)	0.0312 (7)	
C18	0.6527 (4)	0.2806 (2)	0.14923 (10)	0.0309 (7)	
C19	0.9164 (3)	0.98494 (19)	0.21210 (9)	0.0236 (6)	
C20	1.0438 (3)	1.0043 (2)	0.19202 (9)	0.0239 (6)	
C21	0.9078 (3)	0.7118 (2)	0.21691 (9)	0.0251 (6)	
C22	1.0451 (3)	0.74359 (19)	0.23552 (9)	0.0228 (6)	
C23	1.0169 (3)	0.7775 (2)	0.11045 (9)	0.0250 (7)	
C24	0.8931 (4)	0.8354 (2)	0.10380 (9)	0.0259 (7)	
N1	0.2938 (3)	0.43929 (18)	0.10155 (8)	0.0266 (6)	
N2	0.0678 (3)	0.43760 (17)	0.06127 (8)	0.0255 (6)	
N3	0.2680 (4)	0.5798 (2)	0.11054 (9)	0.0376 (7)	
H3A	0.331779	0.582867	0.128817	0.045*	
H3B	0.222891	0.623128	0.102882	0.045*	
N4	0.0990 (3)	0.57149 (19)	0.04458 (9)	0.0357 (7)	
H4A	0.031976	0.570028	0.027065	0.043*	
H4B	0.146263	0.615901	0.048862	0.043*	
N5	0.2674 (3)	0.34121 (17)	0.03123 (8)	0.0257 (6)	
N6	0.0671 (3)	0.25722 (17)	0.05017 (8)	0.0254 (6)	
N7	0.1942 (3)	0.32244 (19)	−0.02999 (8)	0.0305 (6)	
H7A	0.248845	0.359848	−0.037113	0.037*	
H7B	0.140095	0.295671	−0.046000	0.037*	
N8	0.0538 (4)	0.1854 (2)	−0.00403 (9)	0.0364 (7)	
H8A	−0.001209	0.148844	0.003653	0.044*	
H8B	0.079798	0.182109	−0.025952	0.044*	
N9	0.3001 (3)	0.24949 (19)	0.10590 (8)	0.0288 (6)	
N10	0.0988 (3)	0.32704 (17)	0.12955 (7)	0.0237 (5)	
N11	0.2827 (4)	0.1515 (2)	0.15068 (10)	0.0395 (8)	
H11A	0.335046	0.117009	0.140537	0.047*	
H11B	0.247894	0.138823	0.170849	0.047*	
N12	0.1710 (3)	0.2831 (2)	0.18860 (8)	0.0318 (7)	
H12A	0.121272	0.318537	0.199025	0.038*	
H12B	0.222490	0.248919	0.201840	0.038*	
N13	0.3875 (3)	0.91758 (16)	0.21219 (7)	0.0205 (5)	
N14	0.5787 (3)	0.95297 (16)	0.17310 (7)	0.0207 (5)	
N15	0.4270 (3)	1.03191 (19)	0.24762 (8)	0.0341 (7)	
H15A	0.374909	1.014818	0.264004	0.041*	
H15B	0.468299	1.078682	0.250302	0.041*	
N16	0.5712 (3)	1.08961 (17)	0.18838 (8)	0.0273 (6)	
H16A	0.625112	1.104020	0.171938	0.033*	
H16B	0.538972	1.126012	0.202437	0.033*	
N17	0.3809 (3)	0.74086 (15)	0.19129 (7)	0.0205 (5)	
N18	0.6005 (3)	0.80218 (16)	0.22246 (7)	0.0208 (5)	
N19	0.4225 (3)	0.62018 (18)	0.22271 (9)	0.0329 (7)	
H19A	0.359445	0.593874	0.208994	0.040*	
H19B	0.470900	0.595377	0.240508	0.040*	
N20	0.5941 (3)	0.71809 (18)	0.27266 (8)	0.0269 (6)	
H20A	0.656989	0.745600	0.285873	0.032*	
H20B	0.557078	0.675429	0.281707	0.032*	
N21	0.5652 (3)	0.78076 (16)	0.13698 (7)	0.0218 (5)	
N22	0.3539 (3)	0.87233 (17)	0.13046 (7)	0.0228 (5)	
N23	0.4997 (3)	0.72258 (19)	0.08063 (8)	0.0316 (6)	
H23A	0.558307	0.682665	0.083907	0.038*	
H23B	0.445489	0.725997	0.060416	0.038*	
N24	0.3494 (4)	0.8678 (2)	0.06708 (9)	0.0432 (8)	
H24A	0.289947	0.907220	0.063583	0.052*	
H24B	0.381349	0.844116	0.048499	0.052*	
N1H	0.9853 (4)	0.5775 (2)	0.16293 (10)	0.0448 (8)	
N2H	0.2033 (10)	0.0523 (4)	0.0669 (2)	0.126 (3)	
O1	0.3892 (3)	0.44158 (17)	0.13244 (7)	0.0343 (6)	
H1	0.449200	0.406220	0.130726	0.052*	
O2	−0.0262 (3)	0.43808 (16)	0.03019 (7)	0.0347 (6)	
H2	−0.089775	0.405527	0.032868	0.052*	
O3	0.3422 (3)	0.40429 (17)	0.01736 (7)	0.0351 (6)	
H3	0.407626	0.416998	0.032529	0.053*	
O4	−0.0117 (3)	0.19277 (15)	0.06173 (7)	0.0332 (6)	
H4	−0.066035	0.209934	0.076079	0.050*	
O5	0.3719 (3)	0.18831 (17)	0.08945 (8)	0.0417 (7)	
H5	0.436217	0.208659	0.079201	0.063*	
O6	0.0285 (3)	0.38670 (16)	0.14740 (7)	0.0312 (5)	
H6	−0.037500	0.404011	0.133464	0.047*	
O7	0.3165 (2)	0.89258 (14)	0.24155 (6)	0.0253 (5)	
H7	0.255297	0.859568	0.234264	0.038*	
O8	0.6582 (2)	0.98038 (14)	0.14605 (6)	0.0265 (5)	
H8	0.702345	0.941823	0.138398	0.040*	
O9	0.2908 (2)	0.69258 (14)	0.16882 (7)	0.0264 (5)	
H9	0.224378	0.720445	0.160032	0.040*	
O10	0.6920 (2)	0.84692 (14)	0.24630 (6)	0.0263 (5)	
H10	0.746129	0.872557	0.234430	0.039*	
O11	0.6481 (2)	0.71123 (14)	0.14187 (7)	0.0280 (5)	
H11	0.713488	0.720234	0.157638	0.042*	
O12	0.2674 (3)	0.93955 (16)	0.12412 (7)	0.0352 (6)	
H12	0.212831	0.941987	0.140145	0.053*	
O13	0.8061 (3)	0.42528 (16)	0.10084 (7)	0.0322 (5)	
O14	0.5692 (3)	0.43221 (16)	0.06414 (7)	0.0317 (5)	
O15	0.8365 (3)	0.55867 (19)	0.09739 (9)	0.0482 (7)	
O16	0.5786 (3)	0.56575 (18)	0.05959 (8)	0.0438 (7)	
O17	0.7682 (3)	0.33748 (16)	0.03526 (7)	0.0313 (5)	
O18	0.5585 (3)	0.26170 (16)	0.05446 (7)	0.0326 (5)	
O19	0.7289 (3)	0.29564 (18)	−0.02222 (7)	0.0390 (6)	
O20	0.5311 (3)	0.1948 (2)	0.00224 (8)	0.0444 (7)	
O21	0.7961 (3)	0.25263 (16)	0.10273 (7)	0.0305 (5)	
O22	0.5947 (3)	0.33159 (16)	0.12630 (7)	0.0303 (5)	
O23	0.8071 (3)	0.16760 (17)	0.14945 (8)	0.0399 (6)	
O24	0.6293 (3)	0.27345 (19)	0.18052 (7)	0.0427 (7)	
O25	0.8708 (2)	0.91302 (13)	0.20658 (6)	0.0220 (4)	
O26	1.0842 (2)	0.94373 (13)	0.17383 (6)	0.0229 (4)	
O27	0.8682 (3)	1.03459 (15)	0.23084 (7)	0.0326 (6)	
O28	1.0950 (3)	1.07009 (15)	0.19461 (8)	0.0357 (6)	
O29	0.8653 (2)	0.75077 (13)	0.18774 (6)	0.0234 (5)	
O30	1.0915 (2)	0.80565 (13)	0.21976 (6)	0.0223 (4)	
O31	0.8507 (3)	0.65533 (15)	0.22965 (8)	0.0365 (6)	
O32	1.0993 (3)	0.71118 (15)	0.26239 (7)	0.0314 (5)	
O33	1.0756 (2)	0.78179 (14)	0.14334 (6)	0.0244 (5)	
O34	0.8593 (2)	0.87042 (14)	0.13244 (6)	0.0237 (5)	
O35	1.0517 (3)	0.73358 (17)	0.08720 (7)	0.0361 (6)	
O36	0.8374 (3)	0.84520 (17)	0.07336 (7)	0.0358 (6)	
O1W	0.2328 (3)	0.4997 (2)	0.19080 (8)	0.0437 (7)	
O2W	0.4899 (5)	0.4035 (2)	0.21006 (10)	0.0508 (14)	0.797 (12)
O3W	0.6077 (4)	0.5443 (2)	0.15144 (10)	0.0478 (13)	0.840 (11)
O4W	0.7854 (4)	0.4944 (2)	0.20708 (11)	0.0513 (14)	0.835 (11)
O5W	0.4730 (9)	0.0484 (4)	0.0306 (2)	0.099 (3)	0.692 (14)
O6W	−0.1286 (6)	0.0307 (3)	−0.00672 (16)	0.082 (2)	0.878 (14)
O7W	0.9316 (5)	0.0544 (2)	0.10722 (12)	0.0657 (16)	0.909 (13)
O8W	0.7410 (7)	0.9990 (3)	0.05478 (16)	0.083 (2)	0.812 (14)

### Atomic displacement parameters (Å^2^)

**Table:**

	*U*^11^	*U*^22^	*U*^33^	*U*^12^	*U*^13^	*U*^23^
Cr1	0.0252 (3)	0.0352 (3)	0.0237 (3)	0.0008 (2)	0.0050 (2)	0.0045 (2)
Cr2	0.0164 (3)	0.0193 (3)	0.0181 (3)	0.00003 (17)	0.00341 (19)	−0.00098 (18)
Ni1	0.0216 (3)	0.0285 (3)	0.0178 (3)	−0.0004 (2)	0.0037 (2)	0.0016 (2)
Ni2	0.0179 (3)	0.0188 (3)	0.0190 (3)	0.00010 (18)	0.0040 (2)	−0.00044 (18)
C1	0.0309 (17)	0.0331 (18)	0.0228 (16)	−0.0065 (14)	0.0086 (13)	−0.0016 (13)
C2	0.0266 (16)	0.0315 (17)	0.0241 (16)	0.0008 (13)	0.0076 (13)	−0.0001 (13)
C3	0.0225 (15)	0.0303 (16)	0.0222 (15)	0.0026 (13)	0.0045 (12)	0.0005 (12)
C4	0.0254 (16)	0.0281 (16)	0.0234 (16)	0.0028 (13)	0.0036 (13)	0.0016 (12)
C5	0.0206 (15)	0.0369 (18)	0.0304 (17)	0.0010 (13)	0.0044 (13)	0.0082 (14)
C6	0.0197 (15)	0.0347 (17)	0.0207 (15)	−0.0037 (12)	0.0058 (12)	0.0043 (13)
C7	0.0195 (14)	0.0245 (15)	0.0233 (15)	0.0002 (11)	0.0052 (12)	−0.0043 (12)
C8	0.0164 (14)	0.0210 (14)	0.0255 (15)	−0.0003 (11)	0.0038 (11)	−0.0002 (12)
C9	0.0220 (15)	0.0223 (15)	0.0223 (15)	−0.0014 (12)	0.0035 (12)	0.0014 (12)
C10	0.0190 (14)	0.0230 (14)	0.0214 (15)	0.0031 (11)	0.0049 (11)	0.0007 (12)
C11	0.0216 (15)	0.0281 (16)	0.0215 (15)	−0.0005 (12)	0.0054 (12)	0.0002 (12)
C12	0.0218 (16)	0.0336 (18)	0.0231 (16)	0.0023 (13)	0.0038 (13)	0.0056 (13)
C13	0.038 (2)	0.039 (2)	0.0341 (19)	0.0004 (16)	0.0088 (16)	−0.0035 (16)
C14	0.036 (2)	0.039 (2)	0.0306 (18)	0.0057 (16)	0.0126 (15)	0.0027 (15)
C15	0.0288 (18)	0.040 (2)	0.0274 (17)	0.0004 (15)	0.0038 (14)	0.0022 (15)
C16	0.0322 (18)	0.041 (2)	0.0268 (17)	−0.0011 (15)	0.0029 (14)	0.0039 (15)
C17	0.0290 (18)	0.0344 (18)	0.0307 (18)	−0.0021 (14)	0.0053 (14)	0.0023 (14)
C18	0.0301 (18)	0.0355 (18)	0.0281 (17)	−0.0021 (14)	0.0076 (14)	0.0036 (14)
C19	0.0207 (15)	0.0227 (15)	0.0275 (16)	0.0006 (12)	0.0033 (12)	0.0001 (12)
C20	0.0237 (15)	0.0261 (16)	0.0221 (15)	−0.0011 (12)	0.0028 (12)	0.0019 (12)
C21	0.0228 (15)	0.0237 (15)	0.0294 (16)	0.0011 (12)	0.0051 (13)	0.0017 (13)
C22	0.0226 (15)	0.0211 (14)	0.0255 (16)	0.0020 (12)	0.0057 (12)	−0.0023 (12)
C23	0.0234 (16)	0.0283 (16)	0.0239 (16)	0.0000 (12)	0.0053 (12)	0.0005 (13)
C24	0.0249 (16)	0.0284 (16)	0.0247 (17)	−0.0002 (12)	0.0043 (13)	−0.0013 (12)
N1	0.0220 (13)	0.0369 (16)	0.0209 (13)	−0.0046 (11)	0.0011 (10)	−0.0010 (11)
N2	0.0216 (13)	0.0305 (14)	0.0240 (14)	0.0014 (11)	−0.0006 (11)	−0.0003 (11)
N3	0.0438 (19)	0.0357 (17)	0.0326 (16)	−0.0033 (14)	0.0001 (14)	−0.0057 (13)
N4	0.0397 (18)	0.0303 (15)	0.0358 (17)	−0.0006 (13)	−0.0028 (14)	0.0025 (13)
N5	0.0251 (14)	0.0314 (15)	0.0213 (13)	−0.0020 (11)	0.0059 (11)	0.0028 (11)
N6	0.0255 (14)	0.0261 (14)	0.0256 (14)	−0.0019 (11)	0.0074 (11)	−0.0008 (11)
N7	0.0366 (16)	0.0375 (16)	0.0179 (13)	−0.0048 (13)	0.0052 (12)	0.0011 (12)
N8	0.0464 (19)	0.0372 (17)	0.0272 (15)	−0.0112 (14)	0.0118 (14)	−0.0072 (13)
N9	0.0241 (14)	0.0370 (16)	0.0267 (14)	0.0059 (12)	0.0089 (11)	0.0052 (12)
N10	0.0225 (13)	0.0301 (14)	0.0192 (13)	0.0019 (11)	0.0061 (10)	0.0001 (11)
N11	0.0379 (18)	0.0441 (19)	0.0395 (18)	0.0150 (14)	0.0190 (15)	0.0171 (14)
N12	0.0309 (15)	0.0455 (18)	0.0194 (13)	0.0047 (13)	0.0038 (11)	0.0042 (12)
N13	0.0191 (12)	0.0233 (13)	0.0204 (12)	−0.0017 (10)	0.0077 (10)	0.0003 (10)
N14	0.0188 (12)	0.0219 (12)	0.0228 (13)	−0.0003 (10)	0.0090 (10)	0.0000 (10)
N15	0.0401 (17)	0.0330 (16)	0.0323 (16)	−0.0151 (13)	0.0189 (13)	−0.0125 (13)
N16	0.0275 (14)	0.0210 (13)	0.0355 (16)	−0.0016 (11)	0.0138 (12)	−0.0020 (11)
N17	0.0179 (12)	0.0196 (12)	0.0238 (13)	−0.0023 (9)	0.0020 (10)	−0.0026 (10)
N18	0.0182 (12)	0.0227 (13)	0.0214 (12)	−0.0026 (10)	0.0013 (10)	−0.0027 (10)
N19	0.0340 (16)	0.0254 (14)	0.0374 (17)	−0.0055 (12)	−0.0066 (13)	0.0046 (12)
N20	0.0284 (14)	0.0299 (14)	0.0220 (13)	−0.0037 (11)	0.0007 (11)	0.0030 (11)
N21	0.0190 (12)	0.0229 (13)	0.0239 (13)	0.0026 (10)	0.0034 (10)	−0.0032 (10)
N22	0.0193 (12)	0.0263 (13)	0.0231 (13)	0.0041 (10)	0.0033 (10)	0.0037 (10)
N23	0.0314 (15)	0.0387 (16)	0.0243 (14)	0.0042 (13)	0.0009 (12)	−0.0059 (12)
N24	0.048 (2)	0.059 (2)	0.0237 (15)	0.0203 (17)	0.0074 (14)	0.0095 (15)
N1H	0.0413 (19)	0.048 (2)	0.046 (2)	0.0034 (16)	0.0077 (16)	−0.0041 (16)
N2H	0.182 (8)	0.078 (4)	0.116 (6)	0.007 (5)	−0.001 (5)	0.055 (4)
O1	0.0302 (13)	0.0444 (15)	0.0269 (12)	0.0016 (11)	−0.0051 (10)	−0.0034 (11)
O2	0.0310 (13)	0.0386 (14)	0.0322 (13)	−0.0036 (11)	−0.0089 (11)	0.0052 (11)
O3	0.0345 (14)	0.0436 (15)	0.0279 (13)	−0.0124 (11)	0.0059 (10)	0.0059 (11)
O4	0.0374 (14)	0.0323 (13)	0.0323 (13)	−0.0081 (11)	0.0166 (11)	−0.0019 (10)
O5	0.0412 (16)	0.0414 (15)	0.0468 (16)	0.0129 (12)	0.0260 (13)	0.0107 (13)
O6	0.0320 (13)	0.0374 (14)	0.0249 (12)	0.0074 (10)	0.0061 (10)	−0.0044 (10)
O7	0.0255 (11)	0.0298 (12)	0.0219 (11)	−0.0078 (9)	0.0092 (9)	−0.0018 (9)
O8	0.0264 (12)	0.0260 (11)	0.0293 (12)	0.0030 (9)	0.0144 (9)	0.0031 (9)
O9	0.0230 (11)	0.0239 (11)	0.0307 (12)	−0.0012 (9)	−0.0047 (9)	−0.0036 (9)
O10	0.0239 (12)	0.0321 (12)	0.0229 (11)	−0.0084 (9)	0.0025 (9)	−0.0005 (9)
O11	0.0235 (11)	0.0270 (12)	0.0324 (13)	0.0076 (9)	−0.0031 (9)	−0.0071 (10)
O12	0.0360 (14)	0.0395 (14)	0.0326 (13)	0.0188 (11)	0.0160 (11)	0.0135 (11)
O13	0.0290 (13)	0.0367 (13)	0.0311 (13)	−0.0008 (10)	0.0039 (10)	0.0015 (10)
O14	0.0266 (12)	0.0357 (13)	0.0332 (13)	0.0030 (10)	0.0054 (10)	0.0054 (10)
O15	0.0544 (19)	0.0439 (17)	0.0462 (17)	−0.0038 (14)	0.0040 (14)	−0.0021 (13)
O16	0.0491 (17)	0.0387 (15)	0.0443 (16)	0.0104 (13)	0.0087 (13)	0.0014 (12)
O17	0.0290 (13)	0.0429 (14)	0.0224 (12)	−0.0013 (10)	0.0054 (10)	0.0000 (10)
O18	0.0288 (13)	0.0407 (14)	0.0289 (13)	−0.0011 (10)	0.0066 (10)	0.0028 (11)
O19	0.0373 (15)	0.0546 (17)	0.0258 (13)	−0.0103 (12)	0.0065 (11)	0.0000 (12)
O20	0.0449 (17)	0.0532 (18)	0.0359 (15)	−0.0146 (14)	0.0080 (13)	−0.0060 (13)
O21	0.0283 (12)	0.0395 (14)	0.0244 (12)	0.0043 (10)	0.0062 (10)	0.0058 (10)
O22	0.0274 (12)	0.0388 (14)	0.0256 (12)	0.0008 (10)	0.0078 (10)	0.0045 (10)
O23	0.0436 (16)	0.0408 (15)	0.0365 (15)	0.0075 (12)	0.0101 (12)	0.0086 (12)
O24	0.0523 (17)	0.0502 (17)	0.0279 (14)	0.0099 (14)	0.0147 (12)	0.0076 (12)
O25	0.0213 (10)	0.0220 (11)	0.0234 (11)	−0.0009 (8)	0.0063 (8)	−0.0027 (8)
O26	0.0228 (11)	0.0241 (11)	0.0224 (11)	−0.0027 (8)	0.0058 (9)	0.0000 (8)
O27	0.0300 (13)	0.0287 (12)	0.0402 (14)	0.0003 (10)	0.0090 (11)	−0.0107 (11)
O28	0.0443 (15)	0.0268 (13)	0.0379 (14)	−0.0126 (11)	0.0137 (12)	−0.0023 (11)
O29	0.0215 (11)	0.0212 (10)	0.0276 (11)	−0.0025 (8)	0.0024 (9)	0.0005 (9)
O30	0.0220 (11)	0.0225 (11)	0.0224 (11)	−0.0010 (8)	0.0021 (8)	0.0021 (8)
O31	0.0293 (13)	0.0303 (13)	0.0497 (17)	−0.0035 (10)	0.0036 (12)	0.0119 (11)
O32	0.0367 (14)	0.0297 (12)	0.0265 (12)	−0.0004 (10)	−0.0027 (10)	0.0060 (10)
O33	0.0208 (11)	0.0297 (12)	0.0230 (11)	0.0031 (9)	0.0035 (9)	−0.0056 (9)
O34	0.0223 (11)	0.0286 (11)	0.0203 (11)	0.0040 (9)	0.0021 (9)	−0.0008 (9)
O35	0.0383 (14)	0.0417 (15)	0.0289 (13)	0.0100 (11)	0.0063 (11)	−0.0108 (11)
O36	0.0397 (15)	0.0476 (16)	0.0195 (12)	0.0095 (11)	−0.0011 (11)	−0.0033 (10)
O1W	0.0399 (15)	0.0605 (19)	0.0324 (14)	−0.0161 (14)	0.0127 (12)	−0.0054 (13)
O2W	0.068 (3)	0.046 (2)	0.037 (2)	0.0112 (19)	−0.0051 (18)	−0.0030 (16)
O3W	0.046 (2)	0.050 (2)	0.045 (2)	−0.0076 (16)	−0.0051 (16)	0.0077 (16)
O4W	0.056 (3)	0.048 (2)	0.050 (2)	0.0026 (17)	0.0073 (18)	0.0070 (17)
O5W	0.133 (7)	0.065 (4)	0.095 (6)	−0.035 (4)	−0.011 (5)	0.021 (4)
O6W	0.086 (4)	0.056 (3)	0.105 (4)	−0.005 (2)	0.018 (3)	−0.001 (3)
O7W	0.082 (3)	0.053 (2)	0.065 (3)	0.013 (2)	0.023 (2)	0.0124 (19)
O8W	0.102 (5)	0.070 (4)	0.078 (4)	0.005 (3)	0.011 (3)	0.005 (3)

### Geometric parameters (Å, º)

**Table:**

Cr1—O14	1.947 (3)	C17—C18	1.549 (5)
Cr1—O13	1.962 (3)	C18—O24	1.215 (5)
Cr1—O17	1.969 (3)	C18—O22	1.297 (5)
Cr1—O22	1.975 (2)	C19—O27	1.208 (4)
Cr1—O21	1.977 (3)	C19—O25	1.289 (4)
Cr1—O18	1.983 (3)	C19—C20	1.551 (4)
Cr2—O25	1.954 (2)	C20—O28	1.206 (4)
Cr2—O33	1.959 (2)	C20—O26	1.299 (4)
Cr2—O29	1.963 (2)	C21—O31	1.215 (4)
Cr2—O34	1.968 (2)	C21—O29	1.299 (4)
Cr2—O30	1.973 (2)	C21—C22	1.547 (5)
Cr2—O26	1.979 (2)	C22—O32	1.213 (4)
Ni1—N2	2.055 (3)	C22—O30	1.294 (4)
Ni1—N6	2.059 (3)	C23—O35	1.208 (4)
Ni1—N5	2.076 (3)	C23—O33	1.303 (4)
Ni1—N9	2.083 (3)	C23—C24	1.553 (5)
Ni1—N1	2.083 (3)	C24—O36	1.220 (4)
Ni1—N10	2.097 (3)	C24—O34	1.287 (4)
Ni2—N13	2.051 (3)	N1—O1	1.410 (4)
Ni2—N17	2.067 (3)	N2—O2	1.407 (4)
Ni2—N14	2.071 (3)	N3—H3A	0.88
Ni2—N21	2.083 (3)	N3—H3B	0.88
Ni2—N18	2.086 (3)	N4—H4A	0.88
Ni2—N22	2.086 (3)	N4—H4B	0.88
C1—N1	1.291 (5)	N5—O3	1.408 (4)
C1—N3	1.350 (5)	N6—O4	1.415 (4)
C1—C2	1.470 (5)	N7—H7A	0.88
C2—N2	1.295 (5)	N7—H7B	0.88
C2—N4	1.333 (5)	N8—H8A	0.88
C3—N5	1.298 (5)	N8—H8B	0.88
C3—N7	1.329 (4)	N9—O5	1.412 (4)
C3—C4	1.489 (5)	N10—O6	1.413 (4)
C4—N6	1.300 (4)	N11—H11A	0.88
C4—N8	1.322 (5)	N11—H11B	0.88
C5—N9	1.298 (5)	N12—H12A	0.88
C5—N11	1.337 (5)	N12—H12B	0.88
C5—C6	1.478 (5)	N13—O7	1.414 (3)
C6—N10	1.291 (4)	N14—O8	1.408 (3)
C6—N12	1.342 (4)	N15—H15A	0.88
C7—N13	1.294 (4)	N15—H15B	0.88
C7—N15	1.331 (4)	N16—H16A	0.88
C7—C8	1.490 (4)	N16—H16B	0.88
C8—N14	1.297 (4)	N17—O9	1.409 (3)
C8—N16	1.340 (4)	N18—O10	1.412 (3)
C9—N17	1.301 (4)	N19—H19A	0.88
C9—N19	1.324 (4)	N19—H19B	0.88
C9—C10	1.488 (4)	N20—H20A	0.88
C10—N18	1.290 (4)	N20—H20B	0.88
C10—N20	1.338 (4)	N21—O11	1.417 (3)
C11—N21	1.289 (4)	N22—O12	1.412 (3)
C11—N23	1.328 (4)	N23—H23A	0.88
C11—C12	1.489 (5)	N23—H23B	0.88
C12—N22	1.293 (4)	N24—H24A	0.88
C12—N24	1.337 (5)	N24—H24B	0.88
C13—O15	1.217 (5)	O1—H1	0.84
C13—O13	1.312 (5)	O2—H2	0.84
C13—C14	1.541 (6)	O3—H3	0.84
C14—O16	1.199 (5)	O4—H4	0.84
C14—O14	1.306 (5)	O5—H5	0.84
C15—O19	1.226 (5)	O6—H6	0.84
C15—O17	1.290 (5)	O7—H7	0.84
C15—C16	1.542 (5)	O8—H8	0.84
C16—O20	1.215 (5)	O9—H9	0.84
C16—O18	1.295 (5)	O10—H10	0.84
C17—O23	1.223 (5)	O11—H11	0.84
C17—O21	1.292 (4)	O12—H12	0.84
			
O14—Cr1—O13	82.64 (11)	O27—C19—O25	125.5 (3)
O14—Cr1—O17	91.47 (11)	O27—C19—C20	121.0 (3)
O13—Cr1—O17	92.52 (11)	O25—C19—C20	113.6 (3)
O14—Cr1—O22	93.18 (11)	O28—C20—O26	127.1 (3)
O13—Cr1—O22	91.98 (11)	O28—C20—C19	120.0 (3)
O17—Cr1—O22	173.90 (11)	O26—C20—C19	113.0 (3)
O14—Cr1—O21	174.18 (11)	O31—C21—O29	126.3 (3)
O13—Cr1—O21	94.01 (11)	O31—C21—C22	120.3 (3)
O17—Cr1—O21	93.44 (11)	O29—C21—C22	113.4 (3)
O22—Cr1—O21	82.14 (10)	O32—C22—O30	125.8 (3)
O14—Cr1—O18	93.64 (11)	O32—C22—C21	120.6 (3)
O13—Cr1—O18	172.69 (11)	O30—C22—C21	113.6 (3)
O17—Cr1—O18	81.25 (11)	O35—C23—O33	125.3 (3)
O22—Cr1—O18	94.52 (11)	O35—C23—C24	122.2 (3)
O21—Cr1—O18	90.21 (11)	O33—C23—C24	112.5 (3)
O25—Cr2—O33	174.63 (10)	O36—C24—O34	125.5 (3)
O25—Cr2—O29	91.00 (9)	O36—C24—C23	120.3 (3)
O33—Cr2—O29	91.63 (10)	O34—C24—C23	114.2 (3)
O25—Cr2—O34	92.86 (10)	C1—N1—O1	112.1 (3)
O33—Cr2—O34	82.31 (10)	C1—N1—Ni1	115.8 (2)
O29—Cr2—O34	93.49 (10)	O1—N1—Ni1	127.8 (2)
O25—Cr2—O30	91.60 (10)	C2—N2—O2	109.5 (3)
O33—Cr2—O30	93.40 (10)	C2—N2—Ni1	115.4 (2)
O29—Cr2—O30	81.99 (9)	O2—N2—Ni1	125.5 (2)
O34—Cr2—O30	173.71 (10)	C1—N3—H3A	120.0
O25—Cr2—O26	81.77 (9)	C1—N3—H3B	120.0
O33—Cr2—O26	96.01 (10)	H3A—N3—H3B	120.0
O29—Cr2—O26	171.07 (10)	C2—N4—H4A	120.0
O34—Cr2—O26	92.11 (10)	C2—N4—H4B	120.0
O30—Cr2—O26	92.92 (9)	H4A—N4—H4B	120.0
N2—Ni1—N6	95.09 (12)	C3—N5—O3	109.4 (3)
N2—Ni1—N5	87.03 (11)	C3—N5—Ni1	114.8 (2)
N6—Ni1—N5	76.71 (11)	O3—N5—Ni1	126.2 (2)
N2—Ni1—N9	172.73 (12)	C4—N6—O4	110.5 (3)
N6—Ni1—N9	90.29 (12)	C4—N6—Ni1	116.7 (2)
N5—Ni1—N9	98.98 (11)	O4—N6—Ni1	129.6 (2)
N2—Ni1—N1	76.20 (12)	C3—N7—H7A	120.0
N6—Ni1—N1	168.70 (12)	C3—N7—H7B	120.0
N5—Ni1—N1	95.42 (11)	H7A—N7—H7B	120.0
N9—Ni1—N1	99.03 (13)	C4—N8—H8A	120.0
N2—Ni1—N10	97.90 (11)	C4—N8—H8B	120.0
N6—Ni1—N10	99.13 (11)	H8A—N8—H8B	120.0
N5—Ni1—N10	173.87 (12)	C5—N9—O5	110.1 (3)
N9—Ni1—N10	76.38 (11)	C5—N9—Ni1	115.0 (2)
N1—Ni1—N10	89.31 (11)	O5—N9—Ni1	127.2 (2)
N13—Ni2—N17	95.42 (10)	C6—N10—O6	110.9 (3)
N13—Ni2—N14	76.50 (10)	C6—N10—Ni1	114.6 (2)
N17—Ni2—N14	169.69 (11)	O6—N10—Ni1	125.9 (2)
N13—Ni2—N21	172.83 (11)	C5—N11—H11A	120.0
N17—Ni2—N21	89.29 (11)	C5—N11—H11B	120.0
N14—Ni2—N21	99.39 (10)	H11A—N11—H11B	120.0
N13—Ni2—N18	86.67 (11)	C6—N12—H12A	120.0
N17—Ni2—N18	76.06 (10)	C6—N12—H12B	120.0
N14—Ni2—N18	96.84 (11)	H12A—N12—H12B	120.0
N21—Ni2—N18	99.75 (11)	C7—N13—O7	110.3 (2)
N13—Ni2—N22	97.94 (11)	C7—N13—Ni2	116.7 (2)
N17—Ni2—N22	98.42 (11)	O7—N13—Ni2	126.31 (19)
N14—Ni2—N22	89.14 (11)	C8—N14—O8	111.1 (2)
N21—Ni2—N22	75.97 (11)	C8—N14—Ni2	117.0 (2)
N18—Ni2—N22	173.19 (11)	O8—N14—Ni2	129.50 (19)
N1—C1—N3	128.9 (3)	C7—N15—H15A	120.0
N1—C1—C2	113.3 (3)	C7—N15—H15B	120.0
N3—C1—C2	117.7 (3)	H15A—N15—H15B	120.0
N2—C2—N4	126.4 (3)	C8—N16—H16A	120.0
N2—C2—C1	113.2 (3)	C8—N16—H16B	120.0
N4—C2—C1	120.4 (3)	H16A—N16—H16B	120.0
N5—C3—N7	126.2 (3)	C9—N17—O9	109.8 (2)
N5—C3—C4	113.4 (3)	C9—N17—Ni2	116.6 (2)
N7—C3—C4	120.4 (3)	O9—N17—Ni2	127.4 (2)
N6—C4—N8	126.3 (3)	C10—N18—O10	110.5 (3)
N6—C4—C3	113.3 (3)	C10—N18—Ni2	116.6 (2)
N8—C4—C3	120.5 (3)	O10—N18—Ni2	127.34 (19)
N9—C5—N11	126.5 (3)	C9—N19—H19A	120.0
N9—C5—C6	113.4 (3)	C9—N19—H19B	120.0
N11—C5—C6	120.1 (3)	H19A—N19—H19B	120.0
N10—C6—N12	127.3 (3)	C10—N20—H20A	120.0
N10—C6—C5	113.3 (3)	C10—N20—H20B	120.0
N12—C6—C5	119.3 (3)	H20A—N20—H20B	120.0
N13—C7—N15	125.1 (3)	C11—N21—O11	109.9 (3)
N13—C7—C8	113.9 (3)	C11—N21—Ni2	115.9 (2)
N15—C7—C8	120.9 (3)	O11—N21—Ni2	126.2 (2)
N14—C8—N16	126.1 (3)	C12—N22—O12	111.3 (3)
N14—C8—C7	113.1 (3)	C12—N22—Ni2	116.4 (2)
N16—C8—C7	120.8 (3)	O12—N22—Ni2	127.8 (2)
N17—C9—N19	126.3 (3)	C11—N23—H23A	120.0
N17—C9—C10	113.4 (3)	C11—N23—H23B	120.0
N19—C9—C10	120.2 (3)	H23A—N23—H23B	120.0
N18—C10—N20	126.1 (3)	C12—N24—H24A	120.0
N18—C10—C9	113.1 (3)	C12—N24—H24B	120.0
N20—C10—C9	120.7 (3)	H24A—N24—H24B	120.0
N21—C11—N23	127.2 (3)	N1—O1—H1	109.5
N21—C11—C12	113.4 (3)	N2—O2—H2	109.5
N23—C11—C12	119.4 (3)	N5—O3—H3	109.5
N22—C12—N24	127.3 (3)	N6—O4—H4	109.5
N22—C12—C11	113.3 (3)	N9—O5—H5	109.5
N24—C12—C11	119.4 (3)	N10—O6—H6	109.5
O15—C13—O13	125.5 (4)	N13—O7—H7	109.5
O15—C13—C14	120.8 (4)	N14—O8—H8	109.5
O13—C13—C14	113.7 (3)	N17—O9—H9	109.5
O16—C14—O14	126.3 (4)	N18—O10—H10	109.5
O16—C14—C13	120.5 (4)	N21—O11—H11	109.5
O14—C14—C13	113.1 (3)	N22—O12—H12	109.5
O19—C15—O17	126.6 (4)	C13—O13—Cr1	114.4 (2)
O19—C15—C16	120.4 (3)	C14—O14—Cr1	115.5 (2)
O17—C15—C16	113.0 (3)	C15—O17—Cr1	115.9 (2)
O20—C16—O18	125.9 (4)	C16—O18—Cr1	115.2 (2)
O20—C16—C15	120.5 (3)	C17—O21—Cr1	114.9 (2)
O18—C16—C15	113.6 (3)	C18—O22—Cr1	114.7 (2)
O23—C17—O21	125.7 (4)	C19—O25—Cr2	116.2 (2)
O23—C17—C18	120.8 (3)	C20—O26—Cr2	115.4 (2)
O21—C17—C18	113.5 (3)	C21—O29—Cr2	115.5 (2)
O24—C18—O22	125.8 (4)	C22—O30—Cr2	115.3 (2)
O24—C18—C17	120.7 (3)	C23—O33—Cr2	115.3 (2)
O22—C18—C17	113.6 (3)	C24—O34—Cr2	114.9 (2)
			
N1—C1—C2—N2	27.1 (4)	N8—C4—N6—O4	5.0 (5)
N3—C1—C2—N2	−151.3 (3)	C3—C4—N6—O4	−173.6 (3)
N1—C1—C2—N4	−153.6 (3)	N8—C4—N6—Ni1	167.0 (3)
N3—C1—C2—N4	28.0 (5)	C3—C4—N6—Ni1	−11.6 (4)
N5—C3—C4—N6	24.0 (4)	N11—C5—N9—O5	5.7 (5)
N7—C3—C4—N6	−154.1 (3)	C6—C5—N9—O5	−174.6 (3)
N5—C3—C4—N8	−154.7 (3)	N11—C5—N9—Ni1	157.5 (3)
N7—C3—C4—N8	27.2 (5)	C6—C5—N9—Ni1	−22.8 (4)
N9—C5—C6—N10	31.4 (4)	N12—C6—N10—O6	4.4 (5)
N11—C5—C6—N10	−148.9 (4)	C5—C6—N10—O6	−173.9 (3)
N9—C5—C6—N12	−147.0 (3)	N12—C6—N10—Ni1	154.2 (3)
N11—C5—C6—N12	32.7 (5)	C5—C6—N10—Ni1	−24.0 (4)
N13—C7—C8—N14	−18.4 (4)	N15—C7—N13—O7	−5.3 (5)
N15—C7—C8—N14	157.9 (3)	C8—C7—N13—O7	170.8 (3)
N13—C7—C8—N16	159.7 (3)	N15—C7—N13—Ni2	−158.6 (3)
N15—C7—C8—N16	−24.0 (5)	C8—C7—N13—Ni2	17.5 (4)
N17—C9—C10—N18	−23.3 (4)	N16—C8—N14—O8	−3.2 (5)
N19—C9—C10—N18	154.8 (3)	C7—C8—N14—O8	174.7 (2)
N17—C9—C10—N20	154.7 (3)	N16—C8—N14—Ni2	−167.3 (3)
N19—C9—C10—N20	−27.2 (5)	C7—C8—N14—Ni2	10.7 (4)
N21—C11—C12—N22	−25.7 (4)	N19—C9—N17—O9	−5.1 (5)
N23—C11—C12—N22	153.0 (3)	C10—C9—N17—O9	172.8 (2)
N21—C11—C12—N24	153.6 (3)	N19—C9—N17—Ni2	−159.6 (3)
N23—C11—C12—N24	−27.7 (5)	C10—C9—N17—Ni2	18.3 (3)
O15—C13—C14—O16	−2.6 (6)	N20—C10—N18—O10	−5.0 (4)
O13—C13—C14—O16	177.9 (3)	C9—C10—N18—O10	172.9 (2)
O15—C13—C14—O14	178.4 (4)	N20—C10—N18—Ni2	−160.7 (3)
O13—C13—C14—O14	−1.1 (5)	C9—C10—N18—Ni2	17.1 (3)
O19—C15—C16—O20	10.7 (6)	N23—C11—N21—O11	−5.3 (5)
O17—C15—C16—O20	−167.7 (4)	C12—C11—N21—O11	173.3 (3)
O19—C15—C16—O18	−170.8 (4)	N23—C11—N21—Ni2	−156.2 (3)
O17—C15—C16—O18	10.8 (5)	C12—C11—N21—Ni2	22.4 (4)
O23—C17—C18—O24	14.1 (6)	N24—C12—N22—O12	−4.8 (5)
O21—C17—C18—O24	−166.2 (4)	C11—C12—N22—O12	174.5 (3)
O23—C17—C18—O22	−166.8 (4)	N24—C12—N22—Ni2	−162.8 (3)
O21—C17—C18—O22	12.9 (5)	C11—C12—N22—Ni2	16.5 (4)
O27—C19—C20—O28	−1.0 (5)	O15—C13—O13—Cr1	−172.8 (3)
O25—C19—C20—O28	179.0 (3)	C14—C13—O13—Cr1	6.7 (4)
O27—C19—C20—O26	178.4 (3)	O16—C14—O14—Cr1	175.9 (3)
O25—C19—C20—O26	−1.6 (4)	C13—C14—O14—Cr1	−5.2 (4)
O31—C21—C22—O32	2.1 (5)	O19—C15—O17—Cr1	170.1 (3)
O29—C21—C22—O32	−177.5 (3)	C16—C15—O17—Cr1	−11.7 (4)
O31—C21—C22—O30	−177.6 (3)	O20—C16—O18—Cr1	173.7 (3)
O29—C21—C22—O30	2.8 (4)	C15—C16—O18—Cr1	−4.6 (4)
O35—C23—C24—O36	−9.0 (5)	O23—C17—O21—Cr1	170.6 (3)
O33—C23—C24—O36	171.5 (3)	C18—C17—O21—Cr1	−9.1 (4)
O35—C23—C24—O34	171.6 (3)	O24—C18—O22—Cr1	168.9 (3)
O33—C23—C24—O34	−7.8 (4)	C17—C18—O22—Cr1	−10.1 (4)
N3—C1—N1—O1	4.4 (5)	O27—C19—O25—Cr2	−176.9 (3)
C2—C1—N1—O1	−173.7 (3)	C20—C19—O25—Cr2	3.1 (3)
N3—C1—N1—Ni1	162.7 (3)	O28—C20—O26—Cr2	178.7 (3)
C2—C1—N1—Ni1	−15.4 (4)	C19—C20—O26—Cr2	−0.6 (3)
N4—C2—N2—O2	6.9 (5)	O31—C21—O29—Cr2	175.6 (3)
C1—C2—N2—O2	−173.9 (3)	C22—C21—O29—Cr2	−4.8 (3)
N4—C2—N2—Ni1	155.2 (3)	O32—C22—O30—Cr2	−179.0 (3)
C1—C2—N2—Ni1	−25.6 (4)	C21—C22—O30—Cr2	0.7 (3)
N7—C3—N5—O3	5.0 (5)	O35—C23—O33—Cr2	−169.5 (3)
C4—C3—N5—O3	−172.9 (3)	C24—C23—O33—Cr2	10.0 (3)
N7—C3—N5—Ni1	153.4 (3)	O36—C24—O34—Cr2	−177.5 (3)
C4—C3—N5—Ni1	−24.6 (4)	C23—C24—O34—Cr2	1.8 (4)

### Hydrogen-bond geometry (Å, º)

**Table:**

*D*—H···*A*	*D*—H	H···*A*	*D*···*A*	*D*—H···*A*
N3—H3*A*···O9	0.88	2.42	2.863 (4)	112
N3—H3*B*···O35^i^	0.88	2.52	3.387 (4)	168
N4—H4*A*···O2^ii^	0.88	2.13	2.811 (4)	134
N4—H4*B*···O19^iii^	0.88	2.21	2.954 (4)	142
N4—H4*B*···O35^i^	0.88	2.65	3.190 (4)	121
N7—H7*A*···O16^iii^	0.88	2.32	3.183 (4)	167
N7—H7*B*···O35^iii^	0.88	2.36	3.198 (4)	159
N7—H7*B*···O36^iii^	0.88	2.58	3.228 (4)	131
N8—H8*A*···O6*W*	0.88	2.34	3.135 (6)	150
N8—H8*B*···O36^iii^	0.88	2.06	2.932 (4)	169
N11—H11*B*···O28^iv^	0.88	2.14	2.913 (4)	146
N12—H12*A*···O7^v^	0.88	2.56	3.170 (4)	128
N12—H12*B*···O32^vi^	0.88	2.18	3.007 (4)	155
N15—H15*A*···O1*W*^vii^	0.88	2.09	2.948 (4)	166
N15—H15*B*···O31^viii^	0.88	2.25	3.057 (4)	152
N15—H15*B*···O32^viii^	0.88	2.34	3.020 (4)	134
N16—H16*A*···O23^ix^	0.88	2.30	3.129 (4)	157
N16—H16*B*···O32^viii^	0.88	2.43	3.295 (4)	167
N19—H19*A*···O1*W*	0.88	2.07	2.912 (4)	159
N19—H19*B*···O27^vi^	0.88	2.08	2.924 (4)	160
N19—H19*B*···O28^vi^	0.88	2.59	3.210 (4)	128
N20—H20*A*···O24^viii^	0.88	2.38	3.215 (4)	158
N20—H20*B*···O27^vi^	0.88	2.52	3.086 (4)	123
N20—H20*B*···O28^vi^	0.88	2.52	3.383 (4)	168
N23—H23*A*···O16	0.88	2.17	2.858 (4)	135
N23—H23*B*···O19^iii^	0.88	2.15	2.983 (4)	159
N24—H24*A*···N2*H*^ix^	0.88	2.57	3.394 (8)	156
N24—H24*B*···O20^iii^	0.88	2.25	3.116 (4)	170
O1—H1···O22	0.84	1.91	2.750 (4)	176
O2—H2···O17^i^	0.84	1.81	2.644 (4)	176
O3—H3···O14	0.84	1.90	2.730 (4)	171
O4—H4···O21^i^	0.84	1.89	2.726 (4)	172
O5—H5···O18	0.84	1.81	2.647 (4)	173
O6—H6···O13^i^	0.84	1.90	2.727 (4)	171
O7—H7···O30^i^	0.84	1.87	2.698 (3)	167
O8—H8···O34	0.84	1.97	2.775 (3)	159
O9—H9···O33^i^	0.84	1.84	2.679 (3)	177
O10—H10···O25	0.84	1.81	2.642 (3)	171
O11—H11···O29	0.84	1.85	2.681 (3)	172
O12—H12···O26^i^	0.84	1.86	2.702 (3)	177
O15···N1*H*			2.737 (5)	
O20···O5*W*			2.740 (7)	
O23···O7*W*			2.810 (5)	
O24···O2*W*			2.840 (5)	
O36···O8*W*			2.797 (6)	
O1*W*···N1*H*^i^			2.856 (5)	
O3*W*···O4*W*			2.705 (6)	
O5*W*···O8*W*^x^			2.817 (11)	
O6*W*···N2*H*^xi^			2.674 (11)	
O6*W*···O6*W*^xi^			2.723 (11)	
O6*W*···O8*W*^iv^			2.779 (8)	
O7*W*···O8*W*^x^			2.729 (8)	

Symmetry codes: (i) *x*−1, *y*, *z*; (ii) −*x*, −*y*+1, −*z*; (iii) −*x*+1, −*y*+1, −*z*; (iv) *x*−1, *y*−1, *z*; (v) −*x*+1/2, *y*−1/2, −*z*+1/2; (vi) −*x*+3/2, *y*−1/2, −*z*+1/2; (vii) −*x*+1/2, *y*+1/2, −*z*+1/2; (viii) −*x*+3/2, *y*+1/2, −*z*+1/2; (ix) *x*, *y*+1, *z*; (x) *x*, *y*−1, *z*; (xi) −*x*, −*y*, −*z*.
